# The Impact of a Western Diet and Resistance Training in a Rat Model of Mammary Cancer

**DOI:** 10.3390/life15020250

**Published:** 2025-02-06

**Authors:** Jessica Silva, Tiago Azevedo, Rita Ferreira, Maria J. Neuparth, Fernanda Seixas, Mário Ginja, Maria J. Pires, Ana I. Faustino-Rocha, José Alberto Duarte, Paula A. Oliveira

**Affiliations:** 1 Centre for the Research and Technology of Agro-Environmental and Biological Sciences (CITAB), University of Trás-os-Montes and Alto Douro (UTAD), 5000-801 Vila Real, Portugal; silva_jessy@hotmail.com (J.S.); tiagoaazevedo99@gmail.com (T.A.); mginja@utad.pt (M.G.); joaomp@utad.pt (M.J.P.); pamo@utad.pt (P.A.O.); 2 Institute for Innovation, Capacity Building and Sustainability of Agri-Food Production (Inov4Agro), UTAD, 5000-801 Vila Real, Portugal; 3 Animal and Veterinary Research Centre (CECAV), Associate Laboratory for Animal and Veterinary Science-AL4AnimalS, UTAD, 5000-801 Vila Real, Portugal; fseixas@utad.pt; 4 Mountain Research Center (CIMO), Associated Laboratory for Sustainability and Technology in Inland Regions (SusTEC), Polytechnique Institute of Bragança (IPB), 5300-253 Bragança, Portugal; 5 LAQV-REQUIMTE, Department of Chemistry, University of Aveiro, 3810-193 Aveiro, Portugal; ritaferreira@ua.pt; 6 Research Center in Physical Activity, Health and Leisure (CIAFEL), Faculty of Sports—University of Porto (FADEUP), 4200-450 Porto, Portugal; mneuparth@hotmail.com; 7 Laboratory for Integrative and Translational Research in Population Health (ITR), 4200-450 Porto, Portugal; 8 Toxicology Research Unit (TOXRUN), University Institute of Health Sciences, CESPU, 4585-116 Gandra, Portugal; 9 Department of Veterinary Sciences, University of Trás-os-Montes and Alto Douro (UTAD), 5000-801 Vila Real, Portugal; 10 Department of Zootechnics, School of Sciences and Technology, University of Évora, 7004-516 Évora, Portugal; 11 Comprehensive Health Research Center (CHRC), University of Évora, 7004-516 Évora, Portugal; 12 Associate Laboratory i4HB—Institute for Health and Bioeconomy, University Institute of Health Sciences—CESPU, 4585-116 Gandra, Portugal; jose.duarte@iucs.cespu.pt; 13 UCIBIO—Applied Molecular Biosciences Unit, Translational Toxicology Research Laboratory, University Institute of Health Sciences (1H-TOXRUN, IUCS-CESPU), 4585-116 Gandra, Portugal

**Keywords:** breast cancer, exercise training, murine model, obesity

## Abstract

This study aimed to investigate the impact of a Western diet and resistance training on cardiac remodeling in a rat model of chemically induced mammary cancer. Fifty-six female Wistar rats were randomly assigned to one of eight experimental groups, evaluating the impact of Western and standard diets, exercise and sedentarism, and the induction of mammary cancer. Mammary cancer was induced via the intraperitoneal administration of *N*-methyl-*N*-nitrosourea (MNU) (50 mg/kg) at seven weeks of age. The resistance training protocol consisted of ladder climbing three times per week for an 18-week period, with a gradual increase in load over time. At the end of the 20-week experimental period, the animals were anesthetized and underwent echocardiography. Subsequently, the animals were euthanized, and organs and visceral adipose tissue (VAT) were collected and analyzed. A histopathological examination was performed on the mammary tumors. The Western diet increased relative VAT and contributed to cardiovascular and tumor-related changes, including an increase in interventricular septum thickness (IVS) and left ventricle posterior wall thickness (LVPW) at end-systole. Exercise reduced fat accumulation, improved cardiac performance, and helped regulate cardiovascular function, as indicated by a higher eccentricity index (EI) in the WD+EX group compared to the WD group. The WD was associated with increased VAT accumulation and initially delayed tumor initiation; however, over time, it contributed to bigger tumor aggressiveness. This diet also delayed tumor initiation but increased LVPW. Exercise, when combined with a WD, accelerated tumorigenesis, malignant transformation and invasiveness, resulted in the higher prevalence of invasive tumors. These findings underscore the complex and potentially compounding effects of diet and exercise on cancer progression.

## 1. Introduction

Mammary cancer represents one of the most prevalent forms of cancer, affecting approximately 2.3 million women in 2022 [1]. Projections indicate a continued rise in incidence, with an estimated 2.63 million by 2030 and 3.28 million by 2050 [1].

Breast cancer-related cardiac changes can be attributed to a number of factors, including the direct impact of cancer and the cardiotoxic effects of several cancer treatments, such as chemotherapy and radiotherapy [2]. Human investigations have demonstrated that cancer-induced cardiotoxicity can cause a variety of cardiovascular problems, including left ventricular dysfunction, alterations in myocardial shape, and heart failure [3]. However, these cardiac alterations in breast cancer patients are complex, and the specific mechanisms remain under investigation [3].

Molecular processes that contribute to these cardiac alterations include oxidative stress, mitochondrial malfunction, inflammation, and apoptosis. Chemotherapeutic drugs, such as anthracyclines, are known to produce reactive oxygen species (ROS), which causes oxidate damage in cardiomyocytes [4]. Furthermore, inflammatory cytokines produced in response to tumor growth or treatment may worsen cardiac damage. These processes combined cause anatomical and functional changes in the heart, including fibrosis, decreased contractility, and left ventricular dysfunction [5].

Lifestyle factors such as diet and exercise have been proven to have a major impact on cancer progression and prognosis, as well as the development of comorbidities [6,7]. The Western diet, characterized by a high intake of processed foods, refined sugars, low fiber content, and saturated fats, has a profound impact on the biology of Wistar rats [8,9]. Studies have shown that prolonged exposure to this diet leads to significant metabolic alterations, including obesity, insulin resistance, and dyslipidemia [9]. Additionally, it can promote systemic inflammation, oxidative stress, and alterations in gut microbiota, which can compromise intestinal barrier integrity and exacerbate metabolic dysfunctions [10]. These changes mimic many aspects of human metabolic syndrome, making Wistar rats a valuable model for studying the adverse effects of the Western diet on health and their role in the development of chronic diseases such as cardiovascular disease, diabetes, and cancer [9].

This study provides novel insights into the potential of Western diet and resistance training as non-pharmacological interventions to mitigate cardiac alterations in a rat model of mammary cancer. According to our knowledge, no studies have previously evaluated the effect of diet and resistance exercise in cardiac damage. By elucidating the interplay between dietary factors, physical activity, and cancer-induced cardiotoxicity, we aim to contribute to the understanding of potential interventions for minimizing the negative cardiac effects of mammary cancer and improving overall health outcomes in patients.

## 2. Materials and Methods

### 2.1. Ethical Considerations

All biosecurity guidelines pertaining to research involving animal models were strictly followed, in accordance with the European Directive 2010/63/EU and National Decree-Law 113/2013. The experimental protocol was approved by the Portuguese Ethics Committee for Animal Experimentation (Direção Geral de Alimentação e Veterinária; under Approval No. 04583) and by an Ethics Review Body (Órgão Responsável pelo Bem-Estar Animal; with reference 834-e-CITAB-2020).

### 2.2. Experimental Design

**Figure 1:**
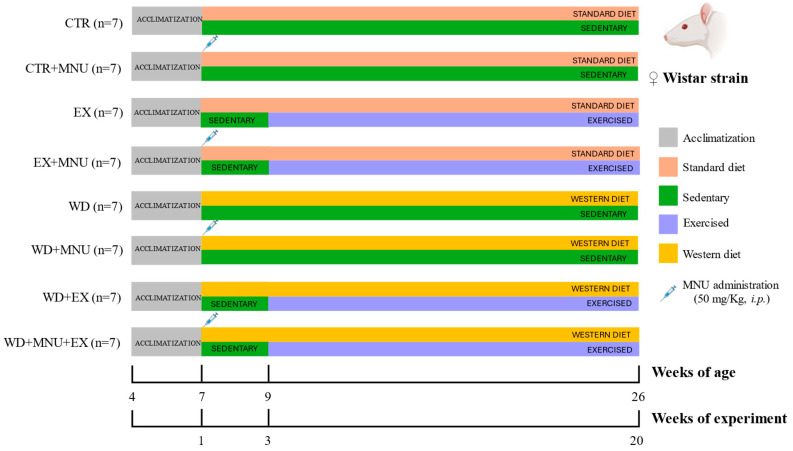
Schematic representation of the experimental protocol. Standard diet and sedentary (CTR); standard diet, sedentary and induced (CTR+MNU); standard diet and exercised (EX); standard diet, exercised and induced (EX+MNU); Western diet and sedentary (WD); Western diet, sedentary and induced (WD+MNU); Western diet and exercised (WD+EX); and Western diet, exercised and induced (WD+MNU+EX).

Fifty-six female Wistar rats (Rattus norvegicus), aged four weeks, were obtained from Envigo RMS Spain S.L. (Barcelona, Spain). The animals were maintained at the Biological Services Unit of the University of Trás-os-Montes and Alto Douro, under carefully controlled conditions of temperature (23 ± 2 °C), humidity (50 ± 10%), air filtration (10–20 ventilations/hour), and a 12 h light/12 h dark cycle. Food and tap water were provided ad libitum. Upon arrival, the animals completed a week of quarantine and two weeks of acclimatization to the lab conditions. Following this period, the animals were randomly divided into eight experimental groups, with each group comprising seven animals (n = 7): standard diet and sedentary (CTR); standard diet, sedentary and induced (CTR+MNU); standard diet and exercised (EX); standard diet, exercised and induced (EX+MNU); Western diet and sedentary (WD); Western diet, sedentary and induced (WD+MNU); Western diet and exercised (WD+EX); and Western diet, exercised and induced (WD+MNU+EX) (Figure 1).

The WD groups (WD, WD+MNU, WD+EX and WD+MNU+EX) were provided with a high-fat diet, in which 60% of the total calories were derived from fat sources, comprising 18.3% from protein, 21.4% from carbohydrates, and 60.3% from fat (MD.06414, Envigo, Spain). The standard diet groups (CTR, CTR+MNU, EX, EX+MNU) were fed with a standard diet (Kcal from: protein 20%, carbohydrate 67% and fat 13%) (2014 Teklad Global Rodent diet, Envigo, Spain).

The animals in the MNU groups (CTR+MNU, EX+MNU, WD+MNU and WD+MNU+EX) were administrated *N*-methyl-*N*-nitrosourea (MNU) intraperitoneally at seven weeks of age, at a dose of 50 mg/kg (Fluorochem, UK), dissolved in saline solution (NaCl 0.9%, B. Braun, Germany) as previously described by Silva et al. [11]. The injection was administered within an hour of preparation, while the non-induced groups received intraperitoneal injections of the vehicle (0.5 mL NaCl 0.9%).

### 2.3. Exercise Training

The exercise training regimen comprised homemade ladder climbing, as previously described [12]. To mitigate the stress associated with the equipment, the animals were acclimated to it by climbing once a day for five consecutive days in the week preceding the start of the training protocol, with no added load. During each session, the animals performed four to eight climbs, each comprising 8–12 dynamic movements, at 80% of their maximal carrying load (MCL). The animals underwent training three days a week for 18 consecutive weeks. The MCL was calculated in accordance with the methodology previously described by Padilha et al. [13]. This test was performed every three weeks, including the last workout session, to determine the point of muscle fatigue and ensure a comprehensive understanding of the animals’ ability to adapt to increasing resistance, providing insights into the effects of diet, exercise, and cancer on muscular endurance and load-bearing capacity.

### 2.4. Animals’ Monitoring

The animals’ health was monitored on a daily basis, and the mammary chains were examined twice a week through palpation by two independent investigators. To determine ponderal weight gain (PWG) and body surface area (BSA), the animals were weekly weighed using a scale (KERN^®^ PLT 6200-2A, Dias de Sousa S.A., Alcochete, Portugal). The formulas described by Paiz et al. (2010) and Li et al. (2020) were used for these measurements [14,15].

### 2.5. Transthoracic Echocardiographic Study

At the end of this study, the anesthesia was prepared by combining xylazine (Rompun^®^ 2%, Bayer S.A., Kiel, Germany) and ketamine (Imalgene^®^ 1000, Merial S.A.S., Lyon, France), at doses of 10 mg/kg and 75 mg/kg, respectively, and injected intraperitoneally. The absence of pedal withdrawal reflexes was verified to assess the depth of anesthesia. Then, the left region of the thoracic wall was shaved using a machine clipper (AESCULAP^®^ Trimmer GT420 Isis, B. Braun, Melsungen, Germany). Each animal was placed in a supine position on a heating pad to maintain its body temperature at 37.0 ± 0.5 °C. The ultrasound gel was preheated and then applied to the thoracic wall, ensuring the absence of air bubbles. Transthoracic echocardiographic examination was performed using the real-time scanner (Logiq P6^®^, General Electric Healthcare, Milwaukee, WI, USA) equipped with a 4–10 MHz linear probe (Model I739, General Electric Healthcare, Milwaukee, WI, USA). Scans of the heart in the parasternal long-axis (PLAX), parasternal short-axis (PSAX), four-chamber, and apical five-chamber views were obtained, using B-mode, M-mode, Color Doppler, and Pulsed Doppler techniques. The images were recorded and analyzed using the MicroDicom 2023.01 viewer software (Sofia, Bulgaria), and the following measurements were taken: the interventricular septum thickness (IVS), the left ventricle internal dimension (LVID), the left ventricle posterior wall thickness (LVPW) at both end-systole and end-diastole, the aorta diameter (Aod), the acceleration time of the pulmonary artery (PAAT), the dimensions of the left (LA) and right (RA) atria, and the left ventricle ejection time (LVET). Additionally, the left ventricle short-axis diameter was measured in two planes: parallel (D1) and perpendicular (D2) to the septum. The measurements were used to determine the fractional shortening of the left ventricle (FS), stroke volume (SV), cardiac output (CO), ejection fraction (EF), left ventricle mass (LV mass), and eccentricity index (EI), using the formulas previously published by our team [16].

### 2.6. Animals’ Euthanasia and Necropsy

Following anesthesia for echocardiography, the animals were then euthanized by exsanguination via cardiac puncture. The heart, VAT, and soleus, gastrocnemius and biceps brachii muscles of each animal were weighed, and the relative organ weights were calculated using the formula described by Santos et al. [17]. The mammary tumors were collected and fixed in 10% buffered formalin for 24 h.

### 2.7. Histopathology of Tumors

All mammary tumors underwent standard histological processing. Three µm thick paraffin sections were cut and stained with hematoxylin and eosin (H&E). Subsequently, a skilled pathologist evaluated the mammary tumors using a light microscope, in accordance with the criteria set by Russo and Russo [18].

### 2.8. Statistical Analysis

The statistical analysis was conducted using SPSS version 26 (Chicago, IL, USA). Data were expressed as mean ± standard deviation (S.D.) for all variables. The Kolmogorov–Smirnov test was used to determine the normality of the data. A multifactorial analysis of variance (ANOVA) followed by the Bonferroni multiple comparisons post hoc test was applied for normally distributed data. The Chi-square test was employed to investigate the relationship between the tumor number, histological tumor type, and group. The Pearson correlation was used to assess the correlation parameters, such as PWG, BSA, relative VAT, LV mass, LVET, µHem, and CK-MB. A *p* value of less than 0.05 was considered statistically significant.

## 3. Results

### 3.1. Animals’ Anthropometric Parameters

Due to a decline in health condition, two animals from the CTR+MNU group and one from the EX+MNU group were euthanized. These animals were excluded from any further analysis. PWG and BSA remained consistent across all groups at the end of this study, indicating that the WD, the exercise regimen, or the induction of mammary cancer did not significantly impact the animals’ body weight (*p* > 0.05, Table 1).

### 3.2. Maximal Carrying Load

**Figure 2:**
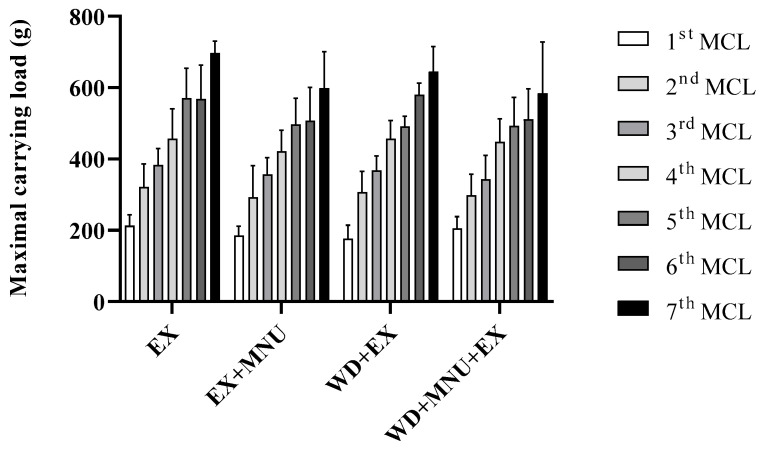
Maximal carrying load (MCL, g) in all exercised groups. Values are presented as mean ± standard deviation (mean ± S.D.). EX: standard diet and exercised control group; EX+MNU: standard diet, exercised and induced group; WD+EX: Western diet and exercised group; and WD+MNU+EX: Western diet, exercised and induced group. Statistically significant differences not found (*p* > 0.05).

Animals in the exercised groups demonstrated an effective adaptation to the exercise regimen (Figure 2). All groups demonstrated a gradual increase in MCL over the course of this study. No statistically significant differences were found among the groups (*p* > 0.05).

### 3.3. Echocardiographic Examination: Morphological Parameters

The data of the echocardiographic examination for the assessment of cardiac morphological alterations are displayed in Table 2. The IVS in end-diastole and end-systole (IVSd and IVSs) was higher in the WD+MNU group when compared with the CTR+MNU group (*p* < 0.05). A reduction in the LVID in the end-diastole and end-systole (LVIDd and LVIDs) was found in the EX+MNU group when compared to the EX group (*p* < 0.05).

No significant differences were observed in the LVPW_d_ among groups (*p* > 0.05). However, the LVPW in end-systole (LVPW_s_) was higher in the WD+MNU group when compared to the CTR+MNU group (*p* < 0.05). The Aod was significantly higher in the EX group when compared to the EX+MNU group (*p* < 0.05) and significantly higher in the WD+MNU group when compared to the CTR+MNU group (*p* < 0.05). No statistically significant differences were identified in the measurement of LA, RA, and D1 among groups (*p* > 0.05). In contrast, the D2 was lower in the WD group when compared with WD+MNU (*p* < 0.05). The LV mass was higher in the EX group when compared with the EX+MNU group (*p* < 0.05).

### 3.4. Echocardiographic Examination: Functional Parameters

The overall assessment of cardiac function included an evaluation of the following parameters: EF, FS, SV, CO, EI, LVET, and PAAT (Table 3). No differences were observed in EF, FS, SV, and CO among groups (*p* > 0.05). The EI was significantly lower in the WD+MNU group compared to the WD group and in the EX group when compared to the WD+EX group (*p* < 0.05). For LVET, a significant decrease was observed in the CTR+MNU group compared to both the EX+MNU (*p* < 0.05) and the WD+MNU groups (*p* < 0.05).

### 3.5. Visceral Adipose Tissue and Organs’ Relative Weight

It was observed that the WD+MNU+EX group exhibited a significantly higher relative heart weight in comparison to the WD+EX group (*p* < 0.05). The WD+MNU group demonstrated a significant increase in relative VAT compared to the WD+MNU+EX group (*p* < 0.05). No statistically significant differences were observed in the relative weight of the soleus, gastrocnemius, and biceps brachii muscles among groups (Table 4).

### 3.6. Correlation Between Data

A statistically significant correlation was identified between anthropometric parameters and cardiac parameters (*p* < 0.05; Table 5). A positive correlation was observed between the BSA and PWG (r = 0.502; *p* < 0.001) and with relative VAT (r = 0.349; *p* = 0.011). Regarding the cardiac parameters, there was a positive and significant correlation between LV mass and LVET (r = 0.416; *p* < 0.001). Statistically significant correlations were not observed among the remaining parameters (*p* > 0.05).

### 3.7. Histological Classification of Mammary Tumors

Animals from the non-induced groups (CTR, EX, WD, WD+EX) did not develop any mammary tumors. The incidence of mammary tumors was similar in the CTR+MNU and EX+MNU groups (3/5, 60% and 4/6, 67%, respectively). The lowest incidence of mammary tumors was observed in the WD+MNU group, with only 29% (2/7), while the highest incidence was observed in the WD+MNU+EX group, with 71% (5/7). Histology revealed that all the tumors were malignant (Table 6). All animals in the CTR+MNU and WD+MNU groups developed invasive tumors, with a total of five and three tumors, respectively (*p* < 0.05). The animals in the EX+MNU group developed seven carcinomas, one carcinoma in situ (cis), and six invasive carcinomas. The WD+MNU+EX group developed a total of fifteen carcinomas, comprising five cis and ten invasive carcinomas.

## 4. Discussion

### 4.1. Effects of Diet and Exercise on Anthropometric Parameters

Cancer is a significant factor influencing body condition, not only due to reduced appetite, but also because of muscle atrophy, decreased fat, and bone mass loss associated with cachexia [19,20]. Conversely, diet and exercise may also change the animal’s body condition [21]. There is the literature reporting that the resistance exercise effectively reduces body weight in animals [22,23,24]; however, other authors have reported that this type of exercise may either have no effect on body weight [25] or even increase it [26,27]. In our study, no differences were observed in PWG or BSA among groups, leading us to believe that neither cancer, nor diet, or intensity of the exercise applied have been sufficient to induce changes in these parameters. Despite this, there was a significant increase in relative VAT in the WD+MNU group compared to the WD+MNU+EX group, suggesting that the WD contributed to fat accumulation and that the exercise mitigated this fat accumulation in animals with cancer [28]. This finding is significant because relative VAT is known to be metabolically active and detrimental to health, contributing to conditions such as cardiovascular disease and even cancer progression. A positive correlation was observed between PWG and BSA, suggesting that an increase in weight, potentially driven by factors such as diet or lack of exercise, is reflected in a larger overall BSA, with greater weight gain corresponding to a larger BSA [29,30]. A positive correlation was also observed between BSA and relative VAT, indicating that animals with a larger BSA tend to have higher amounts of VAT accumulation.

Our results suggest that the WD or resistance exercise did not influence the soleus, gastrocnemius, and biceps brachii masses among groups. No differences were observed in the MCL among groups, with all of them gradually increasing the loads that they were able to support over time, suggesting that neither the cancer, diet, nor resistance exercise influenced the loads that the animals were able to support over time. Despite this lack of differences, an in-depth literature search uncovered no studies that have assessed the effect of ladder resistance training and the Western diet on an animal model of mammary cancer.

### 4.2. Effects of Lifestyle on Mammary Cancer Development

Regarding tumor development, it was observed that both the incidence and the number of tumors *per* animal were slightly higher in the CTR+MNU group (60%; 1.7 tumors *per* animal) compared to the WD+MNU group (29%; 1.5 tumors *per* animal), suggesting that the WD may play a role in suppressing or delaying tumor development. The WD is known to promote obesity and metabolic dysfunction, to which the organism can adapt by activating defense and cellular repair mechanisms that temporarily delay tumorigenesis [31,32]. However, it may contribute to more aggressive cancer progression over time due to the metabolic conditions that it induces, such as chronic inflammation, insulin resistance, hyperglycemia, hormonal imbalance, and leptin and adipokine dysregulation [33,34]. The EX+MNU group exhibited a similar incidence and mean number of tumors *per* animal (67%; 1.8 tumors *per* animal) when compared to the CTR+MNU group (60%; 1.7 tumors *per* animal), suggesting that resistance training may have a minor effect on tumor development in this model. Furthermore, the EX+MNU group showed a broader range of tumor histo-types, including both in situ and invasive carcinomas, indicating that exercise may influence tumor type and potentially its aggressiveness. The WD+MNU+EX group was the group with the highest number of tumors. The mean number of tumors in this group was higher when compared to the WD+MNU group (3.0 tumors *per* animal versus 1.5 tumors *per* animal), with a significant proportion being invasive (10 invasive and 5 in situ), suggesting that resistance exercise, in the presence of a carcinogen in rats fed with a WD, may potentiate tumor development. These results suggest that diet and exercise interact in complex ways to influence tumor development and progression, by increasing blood flow and inadvertently enhance nutrient and oxygen delivery to tumors [32,35].

### 4.3. Effects of Lifestyle on Heart Remodeling

It is noteworthy that the heart relative weight (Table 4) was found to be lower in the WD+EX group than in the WD+MNU+EX group, suggesting that MNU induction increases metabolic demands [36]. Despite these differences, an exhaustive search of the literature revealed no studies that have evaluated the effect of resistance training and mammary cancer on cardiac parameters in rats. The WD+MNU group showed a greater IVS, measured both in systole and diastole, compared to the CTR+MNU group, suggesting that the WD may exacerbate cardiovascular changes in the presence of cancer, possibly due to increased stress on the heart [37]. Similar findings were reported by Maurya et al. (2023) in 8-week-old female C57BL/6 mice fed with a Western diet [38]. Likewise, the WD+MNU group exhibited greater LVPW in systole than the CTR+MNU group, which further indicates that the WD may amplify cardiac alterations in cancer-bearing animals.

Exercise alone (EX group) resulted in a lower EI compared to the WD+EX group, whereas the WD alone (WD group) led to a higher EI compared to the WD+MNU group. This suggests that exercise plays a role in regulating cardiovascular function, even in the presence of a WD. The exercised group (EX group) exhibited significantly larger LVID and Aod compared to the EX+MNU group, suggesting that cancer may limit the heart’s capacity to adapt to exercise [39,40]. It is noteworthy that the EX+MNU group exhibited a longer LVET compared to the CTR+MNU group, which indicates an improvement in cardiac performance in the EX+MNU group. This finding suggests that exercise may help to maintain or even enhance cardiac function despite in the presence of cancer [41,42]. Concerning cardiac parameter correlations, we noticed that there was a negative correlation between LV mass and LVET with CK-MB. This suggests that, in this context, the heart is working harder and taking more time to pump blood, though this increased effort does not necessarily indicate damage. On the other hand, a positive correlation was observed between LV mass and LVET, suggesting that larger hearts tend to require a longer ejection time during systole. This prolonged ejection phase may reflect the increased workload needed for a larger heart to pump blood efficiently. This can reflect either an adaptive or maladaptive response to cardiac stress.

Understanding the complex interaction between lifestyle and factors, such as diet and exercise, and cancer progression may open avenues for developing complementary therapeutic strategies to improve the quality of life and clinical outcomes in cancer patients. However, the lack of significant changes in body weight and muscle mass may suggest that the duration or intensity of the intervention was insufficient to elicit measurable effects in this model. Further investigations are needed to better understand the complex relationship between diet, exercise, and cancer progression, as well as the potential long-term impacts of these factors on tumor behavior and overall health.

## 5. Conclusions

This study underscores the intricate relationship between diet, exercise, and cancer progression, particularly in the context of cardiovascular and metabolic health. The WD promoted the VAT accumulation, which was mitigated by exercise in induced animals. Furthermore, the WD also appeared to delay tumor initiation, potentially due to metabolic adaptations, but eventually led to increased tumor aggressiveness. In rats exposed to a carcinogen, exercise accelerated tumor development under the WD, possibly due to increased nutrients and oxygen delivery to tumors. Resistance training also influenced tumor histological grade, with the WD+MNU+EX group displaying a higher number of invasive tumors, thus emphasizing the complex interactions between exercise, diet, and cancer progression.

The WD exacerbated cardiac remodeling, including increased LVPW, potentially due to higher cardiac stress in cancer-bearing animals. Exercise demonstrated some cardioprotective effects, improving cardiac performance metrics like LVET, even in cancer models. However, cancer appeared to limit the heart’s adaptive capacity to exercise.

## Figures and Tables

**Table 1:** Ponderal weight gain (PWG, %) and body surface area (BSA, cm^2^) of animals from all groups (mean ± S.D.).

Parameter	Experimental Group (n)							
	CTR (n = 7)	CTR+MNU (n = 5)	EX (n = 7)	EX+MNU (n = 6)	WD (n = 7)	WD+MNU (n = 7)	WD+EX (n = 7)	WD+MNU+EX (n = 7)
PWG (%)	48.35 ± 4.24	52.45 ± 2.46	50.85 ± 3.44	47.87 ± 5.58	52.63 ± 6.35	50.12 ± 4.68	52.85 ± 4.74	50.39 ± 2.18
BSA (cm^2^)	388.23 ± 9.74	383.77 ± 28.61	401.13 ± 18.84	370.87 ± 29.00	398.66 ± 23.26	392.12 ± 22.23	396.13 ± 19.23	362.56 ± 27.94

CTR: standard diet and sedentary group; CTR+MNU: standard diet, sedentary and induced group; EX: standard diet and exercised group; EX+MNU: standard diet, exercised and induced group; WD: Western diet and sedentary group; WD+MNU: Western diet, sedentary and induced group; WD+EX: Western diet and exercised group; and WD+MNU+EX: Western diet, exercised and induced group. Statistically significant differences not found (*p* > 0.05).

**Table 2:** Echocardiographic measurements for assessment of cardiac morphological alterations (mean ± S.D.).

Parameter	Experimental group (n)							
	CTR (n = 7)	CTR+MNU (n = 5)	EX (n = 7)	EX+MNU (n = 6)	WD (n = 7)	WD+MNU (n = 7)	WD+EX (n = 7)	WD+MNU+EX (n = 7)
IVS_d_ (mm)	0.85 ± 0.00	0.73 ± 0.03	0.89 ± 0.01	0.78 ± 0.01	1.03 ± 0.01	0.97 ± 0.02 ^a^	0.81 ± 0.01	0.80 ± 0.04
IVS_s_ (mm)	0.79 ± 0.05	0.74 ± 0.15	0.88 ± 0.21	0.82 ± 0.16	0.91 ± 0.21	1.05 ± 0.27 ^a^	0.86 ± 0.15	0.85 ± 0.31
LVID_d_ (mm)	6.75 ± 1.15	6.04 ± 1.79	7.62 ± 0.87 ^b^	5.68 ± 0.90	6.45 ± 1.22	6.01 ± 1.15	7.15 ± 0.64	6.28 ± 2.15
LVID_s_ (mm)	6.30 ± 1.17	5.39 ± 1.71	6.32 ± 1.02 ^b^	4.66 ± 1.22	5.62 ± 1.48	5.35 ± 1.15	6.14 ± 1.03	4.91 ± 1.53
LVPW_d_ (mm)	1.15 ± 1.15	1.03 ± 1.79	1.16 ± 0.87	1.16 ± 0.9	1.12 ± 1.22	1.36 ± 1.15	1.38 ± 0.64	1.05 ± 2.15
LVPW_s_ (mm)	1.31 ± 0.21	0.99 ± 0.32	1.03 ± 0.24	1.18 ± 0.32	1.01 ± 0.13	1.36 ± 0.29 ^a^	1.12 ± 0.16	1.06 ± 0.31
Aod (mm)	1.38 ± 0.19	1.23 ± 0.16	1.62 ± 0.12 ^b^	1.30 ± 0.15	1.58 ± 0.16	1.61 ± 0.20 ^a^	1.49 ± 0.30	1.37 ± 0.48
LA (mm^2^)	3.81 ± 0.40	3.78 ± 1.33	3.71 ± 0.62	3.92 ± 1.32	4.14 ± 0.98	5.33 ± 1.34	4.26 ± 1.21	4.08 ± 1.40
RA (mm^2^)	3.68 ± 0.42	3.34 ± 0.81	4.04 ± 1.09	4.09 ± 1.23	4.33 ± 1.26	5.24 ± 1.52	4.00 ± 0.73	4.49 ± 2.48
D1(mm)	7.52 ± 1.83	9.02 ± 0.61	6.69 ± 1.63	7.72 ± 1.24	6.99 ± 1.35	7.59 ± 1.03	8.03 ± 0.74	7.06 ± 2.27
D2 (mm)	5.73 ± 0.81	7.18 ± 0.67	6.13 ± 1.24	6.97 ± 1.39	4.93 ± 0.77 ^c^	7.26 ± 1.22	5.33 ± 0.45	5.73 ± 2.22
LV mass (mg)	310.81 ± 124.51	228.04 ± 126.82	399.48 ± 154.44 ^b^	231.64 ± 115.50	315.21 ± 120.71	306.72 ± 110.11	386.16 ± 122.19	298.90 ± 125.14

^a^ *p* < 0.05 versus CTR+MNU; ^b^ *p* < 0.05 versus EX+MNU; and ^c^ *p* < 0.005 versus WD+MNU. In the first column, d in subscript means end-diastole, and s means end-systole. IVS: interventricular septum thickness; LVID: left ventricle internal dimension; LVPW: left ventricle posterior wall; Aod: aorta diameter; LA: left atrium dimension; RA: right atrium dimension; D1: left ventricle short-axis diameter parallel; D2: left ventricle short-axis diameter perpendicular; LV mass: left ventricle mass. CTR: standard diet and sedentary group; CTR+MNU: standard diet, sedentary and induced group; EX: standard diet and exercised group; EX+MNU: standard diet, exercised and induced group; WD: Western diet and sedentary group; WD+MNU: Western diet, sedentary and induced group; WD+EX: Western diet and exercised group; and WD+MNU+EX: Western diet, exercised and induced group.

**Table 3:** Echocardiographic measurements and calculations for assessment of cardiac function (mean ± S.D.).

Parameter	Experimental Group (n)							
	CTR (n = 7)	CTR+MNU (n = 5)	EX (n = 7)	EX+MNU (n = 6)	WD (n = 7)	WD+MNU (n = 7)	WD+EX (n = 7)	WD+MNU+EX (n = 7)
EF (%)	30.2 ± 32.55	46.23 ± 32.86	64.77 ± 23.85	68.27 ± 24.33	60.47 ± 19.89	44.89 ± 28.98	58.28 ± 26.10	73.07 ± 23.80
FS (%)	6.65 ± 8.27	10.92 ± 9.49	16.89 ± 10.00	18.92 ± 11.47	13.78 ± 6.94	10.56 ± 9.75	14.52 ± 9.80	20.75 ± 8.16
SV (µL)	4.85 ± 1.12	5.86 ± 0.94	4.47 ± 1.14	3.90 ± 0.44	4.93 ± 1.09	6.51 ± 2.03	4.49 ± 2.49	5.33 ± 0.99
CO (mL/min)	1477.23 ± 445.56	1119.60 ± 652.73	1058.08 ± 246.25	1131.36 ± 353.73	1393.38 ± 420.07	1740.29 ± 953.14	1012.64 ± 634.84	1002.78 ± 425.33
EI	1.31 ± 0.20	1.26 ± 0.11	1.11 ± 0.27 ^b^	1.13 ± 0.20	1.42 ± 0.17 ^a^	1.06 ± 0.19	1.52 ± 0.22	1.19 ± 0.40
LVET (s)	0.07 ± 0.03	0.04 ± 0.04 ^a,c^	0.07 ± 0.01	0.06 ± 0.02	0.07 ± 0.01	0.08 ± 0.01	0.07 ± 0.01	0.07 ± 0.03
PAAT (cm/s)	52.00 ± 4.24	53.25 ± 7.80	56.63 ± 9.80	46.00 ± 9.43	48.00 ± 5.81	47.00 ± 6.05	54.50 ± 5.07	53.67 ± 8.02

^a^ *p* < 0.005 versus WD+MNU; ^b^ *p* < 0.005 versus WD+EX; ^c^ *p* < 0.0001 versus EX+MNU; EF: ejection fraction; FS: fractional shortening of the left ventricle; SV: stroke volume; CO: cardiac output; EI: eccentricity index; LVET: left ventricle ejection time; PAAT: pulmonary artery acceleration time; CTR: standard diet and sedentary group; CTR+MNU: standard diet, sedentary and induced group; EX: standard diet and exercised group; EX+MNU: standard diet, exercised and induced group; WD: Western diet and sedentary group; WD+MNU: Western diet, sedentary and induced group; WD+EX: Western diet and exercised group; and WD+MNU+EX: Western diet, exercised and induced group.

**Table 4:** Relative weight of organs and visceral adipose tissue (mg weight/g body weight) for each experimental group (mean ± S.D.).

Experimental Group (n)	Organ Relative Weight (mg/g)				
	Heart	VAT	Soleus Muscle	Gastrocnemius Muscle	Biceps Brachii Muscle
CTR (n = 7)	2.87 ± 0.16	40.69 ± 7.69	0.74 ± 0.20	11.54 ± 0.86	1.00 ± 0.17
CTR+MNU (n = 5)	2.84 ± 0.16	60.01 ± 11.49	0.72 ± 0.09	10.00 ± 3.76	0.92 ± 0.18
EX (n = 7)	2.71 ± 0.23	38.88 ± 7.30	0.80 ± 0.08	12.16 ± 0.97	1.00 ± 0.12
EX+MNU (n = 6)	2.90 ± 0.50	42.61 ± 15.68	0.78 ± 0.12	11.87 ± 0.88	1.01 ± 0.11
WD (n = 7)	2.62 ± 0.19	53.86 ± 13.61	0.80 ± 0.03	11.80 ± 1.79	0.97 ± 0.09
WD+MNU (n = 7)	2.80 ± 0.20	61.99 ± 12.60 ^a^	0.75 ± 0.06	11.30 ± 0.62	0.98 ± 0.04
WD+EX (n = 7)	2.70 ± 0.20 ^a^	54.36 ± 16.51	0.80 ± 0.09	11.95 ± 0.69	0.96 ± 0.10
WD+MNU+EX (n = 7)	3.24 ± 0.32	39.46 ± 17.01	0.76 ± 0.07	11.87 ± 0.64	0.90 ± 0.11

^a^ *p* < 0.05 versus WD+MNU+EX. VAT: visceral adipose tissue; CTR: standard diet and sedentary group; CTR+MNU: standard diet, sedentary and induced group; EX: standard diet and exercised group; EX+MNU: standard diet, exercised and induced group; WD: Western diet and sedentary group; WD+MNU: Western diet, sedentary and induced group; WD+EX: Western diet and exercised group; and WD+MNU+EX: Western diet, exercised and induced group.

**Table 5:** Correlation between anthropometric parameters (PWG, BSA and relative VAT) and cardiac parameters.

	PWG	BSA	Relative VAT	LV mass	LVET
PWG	-	0.502 ** (*p* < 0.001)	0.234 (*p* = 0.094)	−0.28 (*p* = 0.856)	−0.104 (*p* = 0.458)
BSA	-	-	0.349 * (*p* = 0.011)	0.032 (*p* = 0.834)	0.002 (*p* = 0.989)
Relative VAT	-	-	-	−0.150 (*p* = 0.325)	−0.243 (*p* = 0.083)
LV mass	-	-	-	-	0.416 ** (*p* < 0.001)
LVET	-	-	-	-	-

PWG: ponderal weight gain; BSA: body surface area; VAT: visceral adipose tissue; LV mass: left ventricle mass; and LVET: left ventricle ejection time. * p < 0.05; ** *p* < 0.001.

**Table 6:** Histological classification of each mammary tumor, identified in animals from MNU-induced groups, according to the predominant histological pattern.

Histological Classification		Experimental Group (n)			
		CTR+MNU (n = 5)	EX+MNU (n = 6)	WD+MNU (n = 7)	WD+MNU+EX (n = 7)
In situ	Ductal papillary	0	0	0	4
	Ductal solid and cribriform	0	1	0	1
Invasive	Papillary	1	2	0	1
	Cribriform	4	3	3	9
	Tubular	0	1	0	0
Total number of tumors		5 *	7	3 *	15
Total number of animals with tumors		3	4	2	5
Incidence		60%	67%	29%	71%
Mean number of tumors per animal		1.7 (5/3)	1.8 (7/4)	1.5 (3/2)	3.0 (15/5)

CTR+MNU: standard diet, sedentary and induced group; EX+MNU: standard diet, exercised and induced group; WD+MNU: Western diet, sedentary and induced group; and WD+MNU+EX: Western diet, exercised and induced group. * *p* < 0.05.

## Data Availability

The data that support the findings of this study are available from the corresponding author, [A.I.F.-R.], upon reasonable request.

## Abbreviations

The following abbreviations are used in this manuscript:

VAT	Visceral adipose tissue
IVS	Interventricular septum
LVPW	Ventricle posterior wall thickness
EI	Eccentricity index
CTR	Standard diet and sedentary
CTR+MNU	Standard diet, sedentary and induced
CTR+EX	Standard diet, sedentary and exercised
CTR+MNU+EX	Standard diet, exercised and induced
WD	Western diet
WD+MNU	Western diet and induced
WD+EX	Western diet and exercised
WD+MNU+EX	Western diet, induced and exercised
MNU	*N*-methyl-*N*-nitrosourea
MCL	Maximal carrying load
PWG	Ponderal weight gain
BSA	Body surface area
PLAX	Parasternal long-axis
PSAX	Parasternal short-axis
LVID	Left ventricle internal dimension
Aod	Aorta diameter
PAAT	Acceleration time of the pulmonary artery
LA	Left atria
RA	Right atria
LVET	Left ventricle ejection time
D1	Left ventricle short-axis diameter parallel to the septum
D2	Left ventricle short-axis diameter perpendicular to the septum
FS	Fractional shortening of the left ventricle
SV	Stroke volume
CO	Cardiac output
EJ	Ejection fraction
LV	Left ventricle mass
EI	Eccentricity index
S.D.	Standard deviation
cis	Carcinoma in situ
